# Transfer studies of polystyrene nanoparticles in the *ex vivo* human placenta perfusion model: key sources of artifacts

**DOI:** 10.1088/1468-6996/16/4/044602

**Published:** 2015-07-16

**Authors:** Stefanie Grafmueller, Pius Manser, Liliane Diener, Lionel Maurizi, Pierre-André Diener, Heinrich Hofmann, Wolfram Jochum, Harald F. Krug, Tina Buerki-Thurnherr, Ursula von Mandach, Peter Wick

**Affiliations:** 1 Laboratory for Particles-Biology Interactions, Empa, St. Gallen, Switzerland; 2 Perinatal Pharmacology, Department of Obstetrics, University Hospital Zurich, Zurich, Switzerland; 3 Graduate School for Cellular and Biomedical Sciences, University of Berne, Berne, Switzerland; 4 Powder Technology Laboratory, Ecole Polytechnique Federale de Lausanne, Lausanne, Switzerland; 5 Institute of Pathology, Cantonal Hospital St. Gallen, St. Gallen, Switzerland; 6 Empa, International Research Cooperations Manager, St. Gallen, Switzerland

**Keywords:** nanoparticles, biological barrier, material characterization

## Abstract

Nanotechnology is a rapidly expanding and highly promising new technology with many different fields of application. Consequently, the investigation of engineered nanoparticles in biological systems is steadily increasing. Questions about the safety of such engineered nanoparticles are very important and the most critical subject with regard to the penetration of biological barriers allowing particle distribution throughout the human body. Such translocation studies are technically challenging and many issues have to be considered to obtain meaningful and comparable results. Here we report on the transfer of polystyrene nanoparticles across the human placenta using an *ex vivo* human placenta perfusion model. We provide an overview of several challenges that can potentially occur in any translocation study in relation to particle size distribution, functionalization and stability of labels. In conclusion, a careful assessment of nanoparticle properties in a physiologically relevant milieu is as challenging and important as the actual study of nanoparticle–cell interactions itself.

## 1. Introduction

The field of nanotechnology is growing continuously and nanomedicine is a particular intense research area [1]. To understand the interactions of nanoparticles (NPs) with cells and their biodistribution is a pre-requisite for medical applications such as using NPs as carriers to deliver a drug to a specific target cell or organ. In addition, a better knowledge of the transfer of nanoparticles across external and internal tissue barriers is also of key relevance for the safe design of industrially relevant NPs [2]. It has been shown that the physicochemical properties of NPs determine their fate in biological systems [3, 4]. Different NP characteristics could lead to a selective coating with various biomolecules and thereby inducing distinct cellular responses [5]. Therefore, an extensive particle characterization is indispensable. Researchers suggested analyzing particle size, size distribution, agglomeration state, stability, NP composition, endotoxin contamination, sample purity, batch-to-batch consistency, surface coating and reactivity [6, 7]. In addition, OECD and/or the European Chemicals Agency (ECHA) have specified a comprehensive list of physical chemical endpoints for complete nanomaterial characterization [8, 9]. There is already an increased awareness that these features should be examined not only in water, but also in physiologically relevant media, which are used in the respective experimental set-ups [6, 7]. However, in many cases properties such as dye elution, agglomeration behavior or NP stability *in vivo* cannot be predicted by *in vitro* testing using cell culture media, because the composition of biological fluids outside and inside cells is more complex. In such situations, NP stability has to be assessed directly in the desired biological model.

Here, we want to present a brief case report about the practical challenges which emerged during our investigations of NP transport across the human placenta as an example of a biological barrier tissue. To assess transplacental translocation of NPs and to determine the physicochemical properties responsible for placental transfer, NPs with different characteristics are required. We chose polystyrene (PS) beads as model particles because they are commercially available in numerous sizes and with different surface modifications. The use of such commercial PS beads is widely established and the manufacturers already provide information about the NP characteristics. So, it is assumed that confirmation of such information for one random sample of one supplier is sufficient. Furthermore, to visualize and measure particle concentrations in a biologic environment such as that of the human placenta an easy detectable label is highly convenient. PS beads are available labeled with a range of different fluorescent dyes of high intensity and photostability. We evaluated plain, carboxylate- and amine-modified PS beads with different fluorescent labels to study placental translocation in the human *ex vivo* placental perfusion model.

## 2. Materials and methods

### 2.1. Particles

An overview of all fluorescently labeled PS beads used in this study is provided in table 1. Numbers were assigned to each type of bead according to its functionalization. Yellow-green-labeled non-functionalized (plain) PS beads with sizes of 87 (P1) and 504 nm (P2), and carboxylated PS beads (COOH) with sizes of 100.8 (C1) and 533.8 nm (C2) were purchased from Polysciences Inc. (Warrington, PA, USA). GreenF-labeled, amine-modified (NH_2_) 100 (N1) and 250 nm (N3) PS beads were purchased from micromod Partikeltechnologie GmbH (Rostock, D). Yellow-labeled, amine-modified 170 (N2) and 530 nm (N4) PS beads were purchased from Spherotech (Lake Forest, IL, USA). Envy green-labeled, amine-modified 60 (N5), 250 (N6) and 510 nm (N7) PS beads were customized synthesized from Bangs Laboratories Inc. (Fishers, IN, USA). Orange amine-modified 110 nm (N8) PS beads were purchased from Sigma (St. Louis, MO, USA).

**Table 1.** Summary of PS beads characteristics.

	Plain		Carboxylate-modified		Amine-modified							
	P1	P2	C1	C2	N1	N2	N3	N4	N5	N6	N7	N8
Diameter (nm)^a^	87	504	101	534	100	170	250	530	60	250	510	110
Diameter (nm)^b^ in TEM	78 ± 20	455 ± 32	89 ± 3	499 ± 8	88 ± 7	160 ± 5	224 ± 17	494 ± 29	63 ± 10	181 ± 11	451 ± 28	71 ± 11
Hydrodynamic diameter in DD water (nm)^c^	97 ± 1	445 ± 133	89 ± 1	475 ± 143	n.d.	n.d.	n.d.	n.d.	78 ± 1	202 ± 2	445 ± 143	n.d.
Hydrodynamic diameter in PM (nm)^c^	132 ± 3	621 ± 157	130 ± 1	619 ± 187	n.d.	n.d.	n.d.	n.d.	112 ± 1	328 ± 2	658 ± 9	n.d.
Initial no. of particles/mL in PM^d^	9.55 · 10^10^	4.82 · 10^8^	6.49 · 10^10^	3.66 · 10^8^	6.58 · 10^10^	1.11 · 10^10^	4.02 · 10^9^	3.78 · 10^8^	1.86 · 10^11^	7.66· · 10^9^	4.97 · 10^8^	1.29 · 10^11^
Particle surface (nm^2^)/mL in PM^d^	1.83 · 10^9^	3.14 · 10^8^	1.61 · 10^9^	2.86 · 10^8^	1.62 · 10^9^	8.93 · 10^8^	6.37 · 10^8^	2.89 · 10^8^	2.29 · 10^9^	7.89 · 10^8^	3.17 · 10^8^	2.02 · 10^9^
Detection limit in PM (*μ*g/mL)	<0.078	<0.21	<0.078	<0.039	n.d.	n.d.	<2.5	<0.02	<0.078	<0.078	<0.039	n.d.
Zeta potential in 10 mM NaCl (mV)^b^	−50 ± 9.7	−46.9 ± 4.0	−38.2 ± 10.4	−55.6 ± 4.3	n.d.	n.d.	n.d.	n.d.	−30.2 ± 8.2	−36.2 ± 4.4	−38.9 ± 2.7	n.d.
Zeta potential in PM (mV)^b^	−13.1 ± 7.2	−14.5 ± 6.6	−13.1 ± 10.1	−15.3 ± 8.3	n.d.	n.d.	n.d.	n.d.	−12.7 ± 5.4	−14.1 ± 9.9	−17.3 ± 5.7	n.d.
Fluorescent dye	Yellow green	Yellow green	Yellow green	Yellow green	GreenF	Yellow	GreenF	Yellow	Envy green	Envy green	Envy green	Orange
Dye excitation/ emission (nm)	485/528	485/528	485/528	485/528	475/510	485/520	475/510	485/520	525/565	525/565	525/565	481/644
Number of perfusions	3	3	4	4	1	0	4	3	3	3	3	1

Abbreviations: DD double distilled; PM perfusion medium; TEM transmission electron microscopy; n.d. not determined

a According to the manufacturer’s information.

b Experimentally determined (mean ± SD).

c Experimentally determined (mode ± SD).

d Calculated values.

### 2.2. Particle characterization

The zeta potential of the PS beads in 10 mM sodium chloride and perfusion medium (PM) at pH between 6.8 and 7.2 was determined with a Zetasizer NanoZS (Malvern Instruments, Worcestershire, UK). Particle size distribution of PS beads in double distilled (DD) water and PM was determined by nanoparticle tracking analysis (NTA; NanoSight LM 20 System, software version 2.3.5, NanoSight Ltd, Amesbury, UK) as described previously [10]. The DD water and PM were filtered through a 0.02 *μ*m Anotop® 25 syringe filter (Whatman GmbH, Germany) prior to analysis. For comparison of the different NPs the results were normalized to the area under the NP concentration/size curve.

### 2.3. Stability of fluorescent dye

The detection limit of the fluorescence of PS beads was determined as described previously [11]. To assess the stability of fluorescence the loss of fluorescence intensity was analyzed after incubation of the PS beads in PM at 37 °C for 3, 6, 24, 48 and 72 h using a microplate reader (Biotek Synergy HT, Winooski, VT, USA) with excitation and emission wavelengths as indicated in table 1. The leakage of fluorescence was assessed at the indicated time points during 72 h at 37 °C in PM and in samples from the maternal circulation after 3 and 6 h of placenta perfusion by measuring the fluorescence before and after filtration through a 0.1 *μ*m syringe filter.

### 2.4. Transmission electron microscopy (TEM)

PS bead suspensions as supplied by the manufacturer were applied onto a carbon-coated copper grid and processed for TEM analysis. Images were taken with a Zeiss EM 900 TEM (Zeiss SMT, Oberkochen, Germany) at 80 kV.

### 2.5. Ex vivo human placental perfusion model

The placentas were obtained from uncomplicated term pregnancies after caesarean section at the Department of Obstetrics, Zurich University Hospital. Written informed consent was obtained prior to delivery. The project was approved by the local ethics committee and performed in accordance with the principles of the Declaration of Helsinki. The placenta perfusion was performed as described previously [11, 12]. The PM contained M199 tissue culture medium (Sigma, St. Louis, MO, USA) diluted with Earl’s buffer (dilution 1: 2), 1 g L^−1^ glucose (Sigma, St. Louis, MO, USA), 10 g L^−1^ bovine serum albumin (AppliChem GmbH, Darmstadt, Germany), 10 g L^−1^ dextran 40 (Sigma, St. Louis, MO, USA), 2500 IU/L sodium heparin (B. Braun Medical AG, Melsungen, Germany), 250 mg L^−1^ amoxicillin (GlaxoSmithKline AG, Brentford, UK) and 2.2 g L^−1^ sodium bicarbonate (Merck, Darmstadt, Germany). Placental perfusion was started by adding 25 *μ*g mL^−1^ PS beads into the maternal circulation. The concentration of the PS beads in the fetal and maternal circuit was determined by fluorescence measurement in a microplate reader (Biotek Synergy HT, Winooski, VT, USA). Particle concentrations were corrected for the PM volume in the tubes and volume loss due to sampling before placental transfer was calculated as percentage of transferred PS beads compared to the initially added particle amount.

### 2.6. Fluorescence microscopy

Tissue samples of perfused placentas were fixed in 4% formaldehyde (Formafix AG, Hittnau, Switzerland), dehydrated overnight in a Medite Tissue Processor TPC 15 and embedded in paraffin blocks with the Medite Tissue Embedding System TES 99. Unstained 4 *μ*m thick paraffin sections of perfused placenta were deparaffinized with xylene followed by ethanol 100%. Afterwards the slides were air-dried, covered with VECTASHIELD® Mounting Medium containing DAPI (Vector Laboratories, Burlingame, CA, USA) on a glass slide and the coverslips were sealed with a slide sealant. The slides were analyzed with a Leica DM6000B fluorescence microscope system (Leica Microsystems, Heerbrugg, Switzerland) equipped with a triple band-pass filter set (DAPI/Spectrum Green/Spectrum Orange).

### 2.7. Ninhydrin assay

Next, 20 mM ninhydrin (Fluka, Buchs, Switzerland) solution in ethanol was added to 1 mg mL^−1^ PS^−1^ bead suspension. After 30 min at 80 °C and 300 rpm absorption at 550 nm was measured in a microplate reader (Biotek Synergy HT, Winooski, VT, USA) [13].

### 2.8. Statistical analysis

Data are shown as mean ± standard deviation (SD) from at least three independent experiments. The unpaired Student’s *t*-test was performed using GraphPad Prism software, version 6 (GraphPad Software, La Jolla, CA, USA). Differences were considered statistically significant at a *p*-value below 0.05.

## 3. Results and discussion

NPs were carefully characterized and their properties are displayed in table 1. Some amine-modified PS beads were not completely characterized, because they already showed instability in preliminary experiments and could not be used for further studies. During our investigations we encountered different problems, which did not allow us to conduct a reasonable NP translocation study. A brief overview of the practical challenges during evaluation of the different NP types is provided in table 2 and the topics, particle size, surface modification and charge as well as dye stability, will be discussed extensively.

**Table 2.** Summary of the encountered problems for each PS bead type.

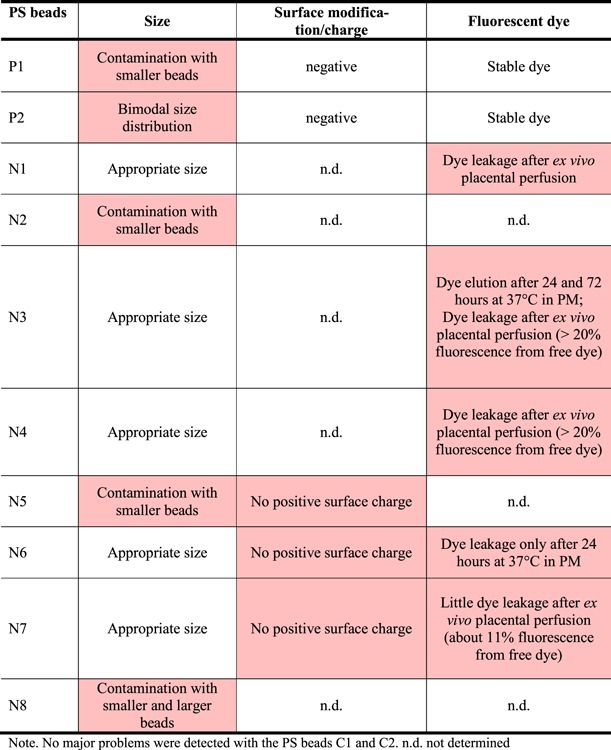

### 3.1. Particle size

**Figure 1.**
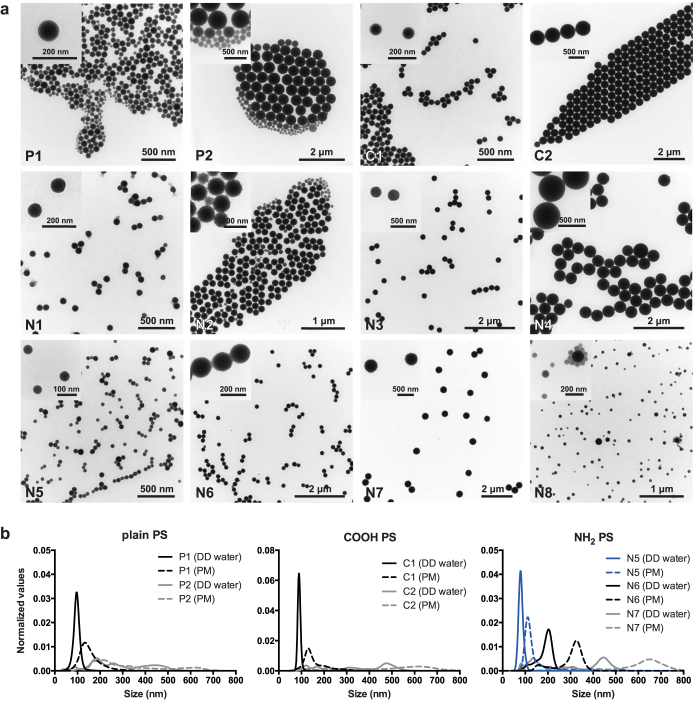
(a) TEM micrographs of PS bead suspensions in DD water with low and high magnification (upper left corner). (b) Size distribution of PS beads in DD water and PM was determined by nanoparticle-tracking analysis.

To unambiguously identify the influence of NP size on placental translocation, a monodisperse suspension is a prerequisite. During evaluation of PS beads for our study, we experienced that obtaining PS beads with a narrow size distribution is quite challenging. Often particle suspensions are contaminated to some extent with beads of a smaller diameter than stated by the manufacturer (figure 1). The TEM micrographs in figure 1(a) illustrate that the PS beads P1, P2, N2, N5 and N8 actually contained particles with various sizes whereas the manufacturer declared one size determined by dynamic light scattering (DLS). DLS measures the average hydrodynamic diameter (*d*) of particles and the intensity of the scattered light is proportional to *d*^6^ (the diameter raised to the power of 6). This term means that a 500 nm particle will scatter 10^6^ times more light than a 50 nm particle. Therefore, DLS measurement is usually biased towards large particles and often prevents the detection of small NPs in polydisperse mixtures. An alternative method for such mixtures is nanoparticle-tracking analysis (NanoSight). This method does not determine the average particle size, but tracks the movement of individual particles in a fluid on a particle-by-particle basis, and thus allows a discrimination of differently sized particles in a polydisperse suspension. However, analyzing particle size distribution by nanoparticle tracking analysis only detected the contamination with smaller beads for larger beads such as P2 (about 500 nm), while PS beads with a diameter around 50 nm such as N5 were already at the detection limit of this method (figure 1(b)). Similar observations that the manufacturer’s size information needs to be confirmed preferably by a complementary technique have been made for other NPs [14].

### 3.2. Surface modification and charge

**Figure 2.**
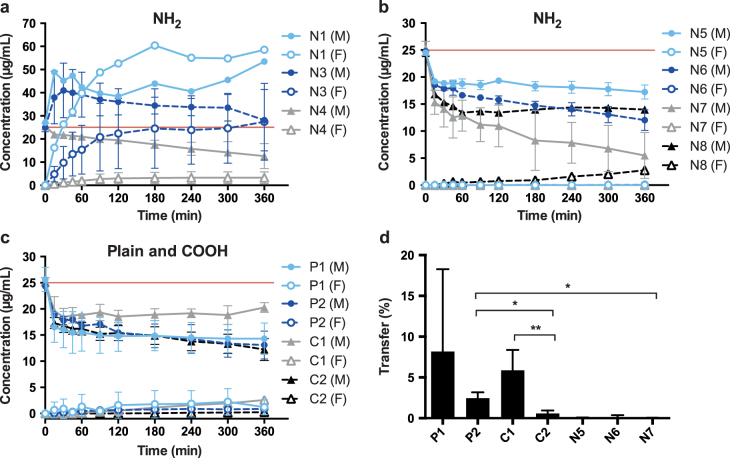
(a), (b) Perfusion profiles after *ex vivo* human placental perfusion of amine-modified PS beads N1 (*n* = 1), N3 (*n* = 4), N4 (*n* = 3), N5, N6, N7 (all *n* = 3), N8 (*n* = 1). (c) Perfusion profiles of plain and carboxylate-modified PS beads P1, P2, C1 and C2 of at least 3 independent experiments. Data are expressed as mean particle concentration (*μ*g mL^–1^) in the fetal (F) and maternal (M) circulation determined by fluorescence measurement at the indicated time points ± SD. The red line indicates the initially added concentration of PS beads (25 *μ*g mL^−1^). (d) Percentage of plain and carboxylate-modified PS beads (P1, P2, C1, C2) in the fetal circulation after 6 h of perfusion compared to the initially added particle amount (mean ± SD of at least 3 independent experiments). (∗) *p* < 0.05, (∗∗) *p* < 0.01.

**Figure 3.**
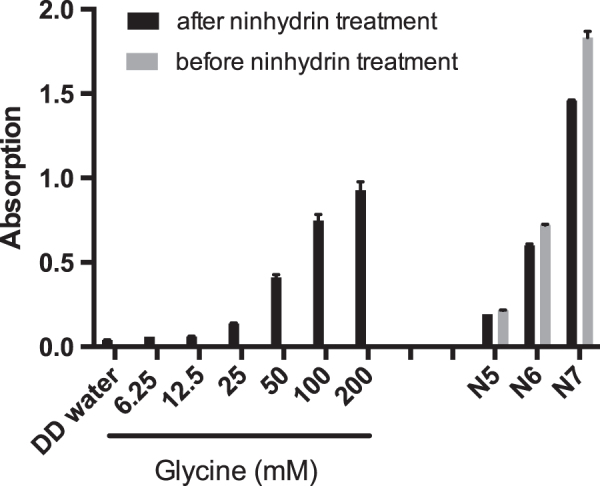
To assess the presence of amino groups on the surface of PS beads N5, N6 and N7, a ninhydrin assay was performed. The amino acid glycine served as positive control. Absorption was determined at 550 nm and mean ± SD of 2 technical replicates is displayed.

Specific particle shells or surface coatings are added to NPs for example to prevent aggregation or to provoke a specific effect *in vivo*. In addition, such coatings are also important parameters by which to induce toxicity or accelerate transport into cells or across barriers [15]. In order to determine the influence of NP surface modification and charge on placental transfer, it is crucial to confirm the presence of these functionalized groups. In *ex vivo* placenta perfusion studies, we observed a difference in placental transport between plain, carboxylate-modified and amine-modified PS beads (figure 2). Plain PS beads (P1 and P2) but not the amine-modified N5, N6 and N7 PS beads were transported across the placental barrier suggesting that the surface modification is a major determinant of placental translocation (figure 2(d)). Zeta potential measurements revealed that, contrary to expectations, these amine-modified NPs were negatively charged in the entire pH range 3–10. Since the fluorescent dye in the particles is slightly negatively charged, it might be that the density of the amine functionalization on the PS beads was not high enough to cause a positive zeta potential. Besides an insufficient functionalization, the particle suspensions could also contain residues of sodium dodecyl sulfate (SDS). SDS was used as surfactant during synthesis of at least some of the used PS beads and is negatively charged. Small residual amounts of SDS in combination with a low amine group density could explain the negative zeta potential. Hence, a direct detection of the chemical amine group would be required, but the attempt to detect the amine groups on the particle surface by the colorimetric ninhydrin assay failed due to NP interference with the assay. Primary amine groups react with ninhydrin and form a purple compound, which leads to an increased absorbance at 570 nm. The internal fluorescence of the PS beads caused a false positive signal and thus, a difference in intensity before and after ninhydrin treatment could not be observed (figure 3). Consequently, the ninhydrin assay is not suitable to confirm the presence of functional amine groups attached to fluorescently labeled NPs and analytical methods such as infrared spectroscopy may be considered for further studies.

### 3.3. *In vitro* and ex vivo elution of fluorescent dyes

**Figure 4.**
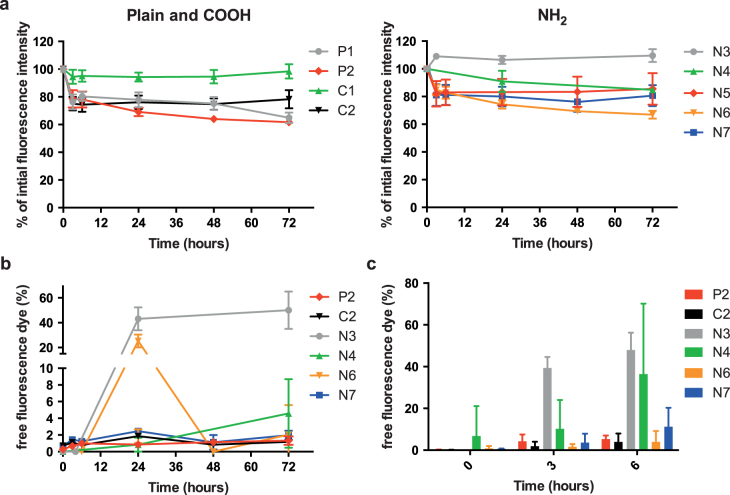
(a) Stability of the fluorescent signal of PS beads in PM at 37 °C over 72 h. The figure displays the percentage of initial fluorescence intensity at several time points compared to the initial fluorescence intensity (mean ± SD; *n* = 3). b) Leakage of the fluorescent dye from the large PS beads (>150 nm) during 72 h at 37 °C in PM. Data are expressed as percentage of free fluorescence compared to the total fluorescence intensity (mean ± SD; *n* = 3). (c) Elution of fluorescent dye of the large PS beads after 3 and 6 h of *ex vivo* placental perfusion. Data are expressed as percentage of free fluorescence compared to the total fluorescence intensity (mean ± SD of at least 2 independent experiments).

Several studies have demonstrated that a fluorescent dye can elute from NPs after contact with biological fluids [16–19]. Therefore, we tested the stability of the fluorescent signal of the PS beads after incubation at 37 °C for up to 72 h in PM. The fluorescence intensity of the greenF-labeled N3 and the yellow-green C1 PS beads remained completely stable over 72 h and only the N3 PS beads showed an elution of fluorescent dye from the particles (43.1% ± 9.3% of total fluorescence intensity was from free fluorescent dye after 24 h) (figure 4(b)). For all other PS beads the fluorescence intensity decreased to 60–80% of the initial signal after 72 h (figure 4(a)). Figure 4(b) indicates that this loss in fluorescence intensity was not due to a significant leakage of fluorescent dye except for the envy-green-labeled N6 PS beads, which showed a transient release of dye after 24 h (25.3% ± 5.2%). Due to technical issues, PS beads with a diameter of 100 nm and below could not be analyzed for fluorescence leakage. Syringe filters with a smaller pore size than 0.1 *μ*m, which are required to separate the free fluorescent dye from the particles, were not applicable because components of the PM led to a clogging of the filter. The overall reduction of the measured fluorescence intensity for most of the PS beads (P1, P2, C2, N4, N5, N6, N7) after incubation in PM is unclear but might be caused by adsorption of albumin or other components of the medium to the NPs, which might impair the fluorescence signal. In addition, unspecific binding of the particles to the tissue culture plastics or particle agglomeration and subsequent sedimentation may explain the overall loss in fluorescence intensity over prolonged exposure in PM.

**Figure 5.**
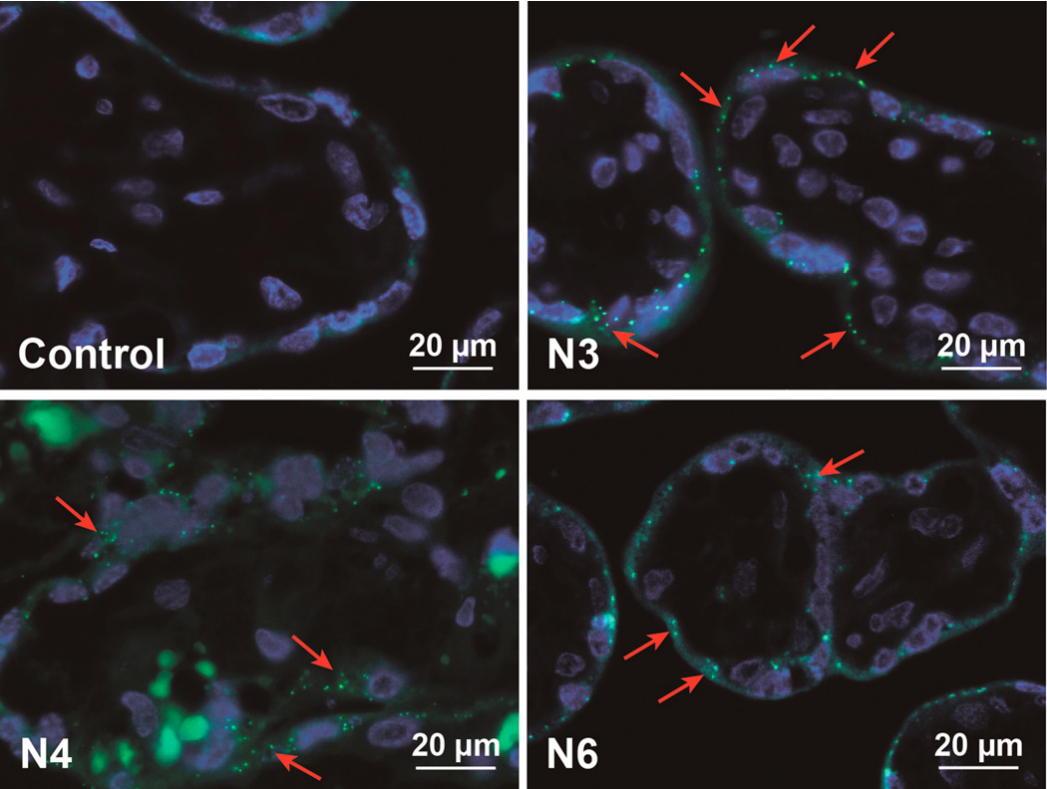
Fluorescence microscopy of placental tissue after 6 h of *ex vivo* perfusion. Displayed are images from representative placenta perfusion experiments with two amine-modified PS beads (N3 and N4), which showed fluorescent dye leakage, and one amine-modified PS particle representative for an NP without fluorescence leakage (N6). As control placental perfusion without addition of particles was performed. Micrographs show PS beads (green) and DAPI-stained nuclei (blue). Due to autofluorescence, erythrocytes appear also in green. Red arrows indicate accumulated PS beads.

Although dye elution was not a major problem for most PS beads after incubation in PM, it became an important issue after contact with the human placenta. During placenta perfusion the greenF-labeled N1 and N3 PS beads showed an unexpected perfusion profile (figure 2(a)). After addition of 25 *μ*g mL^−1^ PS bead into the maternal circulation, the total concentration (fetal and maternal circuit) seemed to increase above the initially added NP concentration. Further analysis of samples from the fetal and maternal circulation after 6 h of perfusion revealed that 47.9% ± 8.3% of the fluorescent dye was leaking out of the greenF-labeled N3 PS particles (figure 4(c)). Although the perfusion profile of the yellow-labeled N4 PS particles appeared more rational, the filtration also showed 36.4% ± 33.8% leakage of the fluorescent dye after 6 h of perfusion (figures 4(c) and 2(a)). According to the particle supplier, the fluorescence in the particles is partially quenched because of the heavy dye loading; thus we suggest that the perfusion profile showed an increased fluorescence in the maternal circuit due to the fact that the free dye generates a higher fluorescence. Consequently, the increased fluorescence intensity in the fetal circulation was likely due to the rapid transfer of the free dye across the placental barrier. GreenF and yellow are fluorescein derivatives and it has been shown that such small hydrophobic chemical compounds can easily cross the placenta [20]. Regardless of the fluorescence leakage, analysis of histological sections of placental tissue after perfusion by fluorescence microscopy still showed fluorescent particles in the syncytiotrophoblast and the villous mesenchyme (figure 5). We were not able to detect a difference in fluorescence distribution within the cells as described previously for rhodamine labeled N-isopropylacrylamide (NIPAM) NPs. These NPs were found to elute dye and the free dye was spread in the whole cell and particle-bound dye was only restricted to organelles such as lysosomes [18]. According to the NP suppliers the water insoluble fluorescent dyes were incorporated in the glassy polymer matrix after swelling in an appropriate solvent and are not chemically bound in the particle or to the surface. Therefore all dyes should remain entrapped in an aqueous environment. However, as in every biological environment, the placental tissue consists of several compartments with different pH values and contains different proteins or lipids. It is known that dyes can elute from NPs after contact with a hydrophobic milieu in a cell [16, 17]. The plasma membrane of a cell retains an enormous hydrophobic area which is especially enlarged in the human placenta compared to other organs. The brush border membrane of the placental syncytiotrophoblast, which is in contact with the maternal blood, forms numerous microvilli to gain a huge surface for effective exchange of nutrients and waste products [21]. Hence, the possibility of NP contact with the hydrophobic membrane in the placenta is much higher than in other biological tissues and therefore the probability of dye elution is increased. Of note, the placenta is an organ with an increased enzymatic and metabolic activity due to its physiological function to protect the fetus and detoxify harmful substances [22, 23]. So it could also be conceivable that parts of the greenF and yellow amine-modified PS particles were degraded by the placental cells which led to the release of the fluorescent dye. Since this phenomenon could not be observed with the non-functionalized and carboxylate-modified PS beads this fluorescence leakage seems to be dependent on the surface modification. However, examinations with amine-modified N5, N6, N7 and N8 PS beads containing a different fluorescent dye (envy green and orange) revealed a fraction of only 3.9% ± 5.2% and 11.2% ± 9.1% free dye after 6 h of perfusion for N6 and N7 nm PS beads, respectively, indicating that dye elution depends mainly on the properties of the fluorescent dye (figures 4(c) and 2(b)).

In conclusion, our study shows that a careful assessment of NP size distribution, modification and fluorescent dye leakage under physiological conditions is essential to prevent artifacts in placental translocation studies using fluorescent PS beads. However, additional NP properties may become relevant for other types of NPs. For soluble NPs such as various metal oxides, it is important to understand particle dissolution to be able to distinguish between uptake and/or translocation of NPs, particle fragments or dissolved ions. A recent NP translocation study in rats demonstrated that it is of key relevance to perform such NP dissolution studies under *in vivo*-like conditions [24]. In this work, the fast clearance of inhaled BaSO_4_ NPs did not correlate with its very low dissolution rate in phagolysosomal simulant fluid (PSF) [24], a proposed model of macrophage dissolution/clearance of particles. The authors conclude that the currently used cell-free *in vitro* dissolution studies either lack crucial constituents or do not adequately simulate the processes that facilitate particle dissolution and increase bioavailability. Another important characteristic in particular for engineered nanoparticles is particle agglomeration. A recent review identified that NP agglomeration affects translocation across tissue barriers as well as uptake and intracellular distribution within the cells [25].

## 4. Conclusions

From the current literature it appears that NP translocation studies across biological barriers are easy to design and relatively straightforward to perform. Here, we provide for the first time an extensive overview of important practical challenges that can occur in such studies using fluorescently labeled PS NPs. A careful validation of NP characteristics such as size distribution and fluorescent dye leakage after contact with biological fluids and stability of surface modifications is crucial in order to obtain meaningful results. To understand how NP characteristics such as size, surface charge and chemistry determine placental transport, cellular responses and uptake mechanisms their physicochemical properties have to be carefully analyzed in physiologically relevant conditions in the selected biological systems. Despite the increasing commercial availability of various NPs with distinct characteristics, the extensive re-characterization of NP properties is indispensable and requires a similar effort as the investigation of the nanoparticle–cell interactions itself. Hence, such studies are a real interdisciplinary challenge.
